# Foraging Behaviour in Magellanic Woodpeckers Is Consistent with a Multi-Scale Assessment of Tree Quality

**DOI:** 10.1371/journal.pone.0159096

**Published:** 2016-07-14

**Authors:** Pablo M. Vergara, Gerardo E. Soto, Darío Moreira-Arce, Amanda D. Rodewald, Luis O. Meneses, Christian G. Pérez-Hernández

**Affiliations:** 1 Universidad de Santiago de Chile, Laboratorio de Ecología y Conservación, Departamento de Gestión Agraria, Libertador Bernardo O’Higgins 3363, Santiago, Chile; 2 Cornell University, Cornell Lab of Ornithology and Department of Natural Resources, Ithaca, NY, United States of America; 3 Universidad de Chile, Laboratorio de Conservación, Departamento de Ciencias Ecológicas, Facultad de Ciencias, Santiago, Chile; Arizona State University, UNITED STATES

## Abstract

Theoretical models predict that animals should make foraging decisions after assessing the quality of available habitat, but most models fail to consider the spatio-temporal scales at which animals perceive habitat availability. We tested three foraging strategies that explain how Magellanic woodpeckers (*Campephilus magellanicus*) assess the relative quality of trees: 1) Woodpeckers with local knowledge select trees based on the available trees in the immediate vicinity. 2) Woodpeckers lacking local knowledge select trees based on their availability at previously visited locations. 3) Woodpeckers using information from long-term memory select trees based on knowledge about trees available within the entire landscape. We observed foraging woodpeckers and used a Brownian Bridge Movement Model to identify trees available to woodpeckers along foraging routes. Woodpeckers selected trees with a later decay stage than available trees. Selection models indicated that preferences of Magellanic woodpeckers were based on clusters of trees near the most recently visited trees, thus suggesting that woodpeckers use visual cues from neighboring trees. In a second analysis, Cox’s proportional hazards models showed that woodpeckers used information consolidated across broader spatial scales to adjust tree residence times. Specifically, woodpeckers spent more time at trees with larger diameters and in a more advanced stage of decay than trees available along their routes. These results suggest that Magellanic woodpeckers make foraging decisions based on the relative quality of trees that they perceive and memorize information at different spatio-temporal scales.

## Introduction

Theoretical models of habitat selection and optimal foraging predict that animals should make foraging decisions, such as patch selection and patch residence time, based on information about resources within “available” habitat patches [1], [2]. Depending on the accuracy and amount of information gathered, foragers should select and remain in high quality patches until their instantaneous gain rate falls to the average rate for all patches available [3–5]. Such decisions assume that foragers consider the potential fitness benefit (e.g., the harvest rate) of using available patches while also considering the costs associated with the presence of conspecifics, competitors and predators [6–8].

The value of information to a foraging animal may vary temporally and spatially. Within their home ranges, animals can use long-term memory to return to high quality sites, though this is often supplemented by short-term and genetically-coded memory [9–15] as well as perceptual and social cues (e.g., [16–18]). Despite having the ability to evaluate habitat quality via different cues and sources of information, wild animals rarely behave as omniscient foragers [19]. The ability to make optimal decisions may be especially compromised in complex and/or variable habitats, which can require animals to navigate throughout unfamiliar patches while being imperfectly informed about the quality of the previously-visited patches [5, 20, 21]. In these complex and/or variable habitats, individuals face important knowledge-related challenges. First, the information an animal uses to make foraging decisions may be unreliable if the food content of patches varies unpredictably over time [22–24], resulting in animals that opportunistically search for randomly distributed resources [25]. Second, insufficient exploration and memory decay can impair the ability to recognize spatial heterogeneity in habitat quality, and this is especially likely for animals whose home ranges include a large number of patches [15, 22, 24–27]. Perceptions and sensitivity to high travel costs may discourage foragers from exploring isolated but high quality patches, with reluctance to travel generally increasing with predation risk [6, 28–30]. Consequently, the uncertainty associated with foraging decisions will rise with increasing variation in habitat quality and declining ability of animals to recognize this variability.

Here, we address the spatio-temporal scales at which Magellanic woodpeckers (*Campephilus magellanicus*) assess the relative quality of trees. Magellanic woodpeckers are specialized to feed on larvae of wood-boring insects that live and promote decay in the bole or branches of beech trees (i.e., belonging to the *Nothofagus* genus) [31–37]. Previous studies suggest that Magellanic woodpeckers feed preferentially on standing trees that exhibit an advanced decay stage [32, 34, 38, 39]. These preferences are consistent with natural and experimental studies that demonstrate the ability of woodpeckers to recognize the type and amount of food (e.g., larvae) present in trees (e.g., [40–42]). Although theoretical studies suggest that foraging woodpeckers respond perceptually and cognitively to habitat heterogeneity over different spatial scales [43], the spatio-temporal scales at which woodpeckers assess the tree quality in the wild remains to be determined.

**Fig 1:**
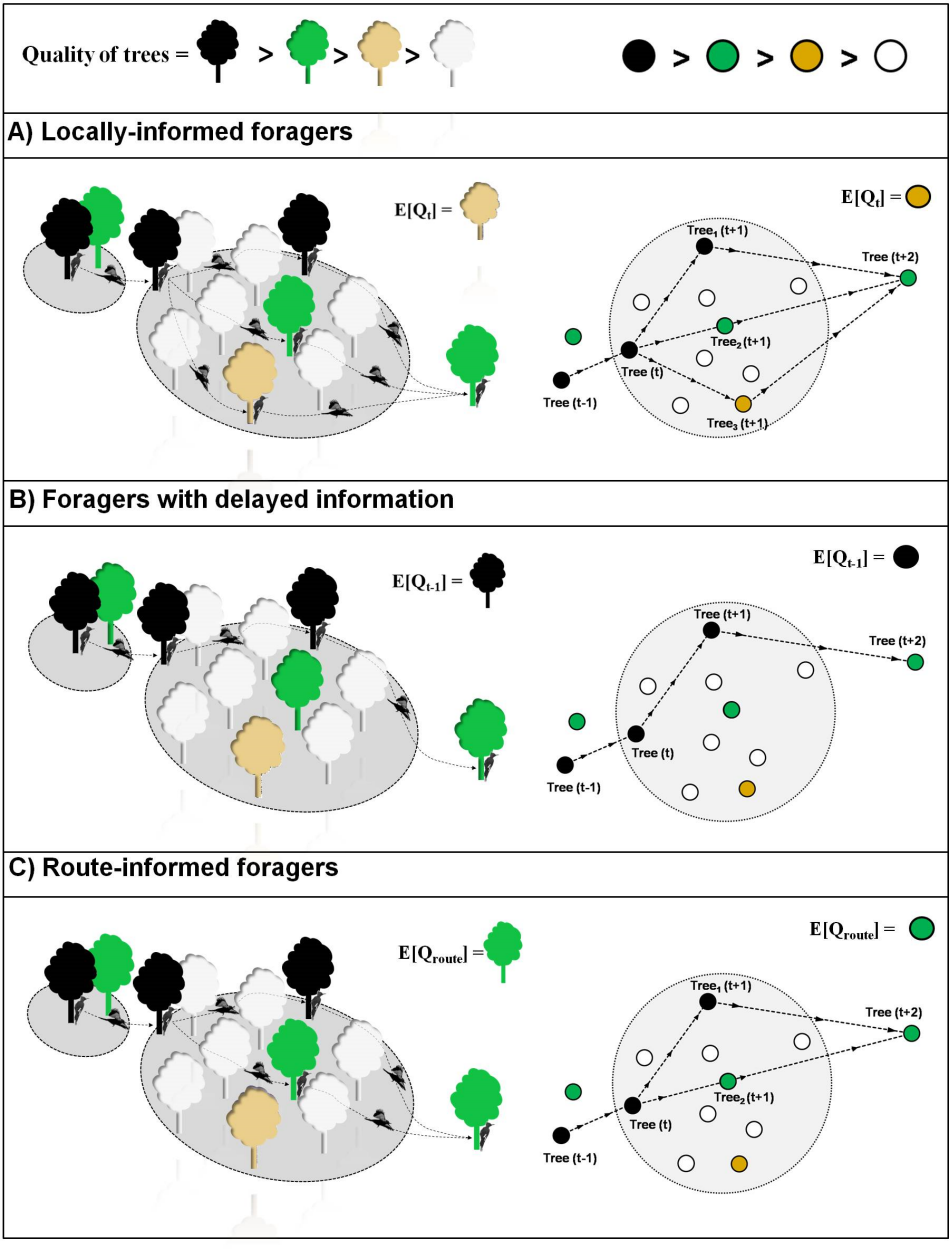
Magellanic woodpeckers behaving according to three types of foraging strategies represented in panels A, B and C (see the main text), including a three- and two-dimensional view of the same route followed by a woodpecker, as predicted under each foraging strategy (i.e., the 2D view makes it easier to interpret the movement pattern). These diagrams highlight the possible trees that the woodpecker could select during period *t*+1 from a set of available trees (n = 9). Available trees are contained within an “availability” circle, which is defined as the 90% isopleth for a circular distribution generated from a Brownian Bridge Movement Model (BBMM; see the Methods section). Tree selection depends on quality expectations for available trees *E*(*Q*). A tree is likely to be selected during period *t*+1 only if the condition *E*(*Q*_*tree*_) ≥ *E*(*Q*) is true. Differences among foragers are highlighted through contrasting values of *E*(*Q*). Locally informed foragers (A) may choose among three possible routes since all of them satisfy the condition *E*(*Q*_*tree*_) ≥ *E*(*Q*). Foragers with delayed information (B) base their movements on the high quality trees contained in the availability circle of the period t– 1, which restrict their movement to only one possible route. Route-informed foragers (C), here representing the intermediate case, may choose between two possible routes through trees whose quality is similar or better than that expected for trees present along the complete route.

Like other woodpecker species, Magellanic woodpeckers forage by sequentially visiting trees within forest stands ([32]; see also [44, 45]). Under theoretical expectations and within foraging activities, each time a woodpecker decides to move to another tree, the quality of the tree selected during the next period *t*+1 should be higher than the expected quality for available trees during the current period *t*. Here, we examined evidence for use of three alternative foraging strategies that differ in how Magellanic woodpeckers process and use information across different spatio-temporal scales:

1. *Locally-informed foragers* (*LF*) are knowledgeable about the trees in their immediate vicinity (i.e., trees within their perceptual range) while using local information to select the next tree (Fig 1A). Such short-term, fine-scale habitat selection is reflected in models addressing the dynamic resource utilization pattern exhibited by animals while moving between consecutive locations [46–50]. However, if trees vary temporally and spatially in quality, as typically faced by woodpeckers within their home ranges (e.g., [51, 52]), they should be less able to make behavioral adjustments based on local habitat conditions. Instead, as hypothesized below, woodpeckers could use a behavioral rule that incorporates information about the condition of trees available over different periods and spatial scales (Fig 1B and 1C).
2. *Delayed-informed foragers* (*DF*) make decisions based on the trees available near previously visited trees, using delayed information (i.e., during *t*-1, *t*-2, *t*-3…*t*-Δ periods, with Δ being the lag period at which the woodpecker assesses quality of available trees). Thus, depending on how tree quality varies spatially, *DF* woodpeckers can select considerably different trees from those selected by a *LF* woodpecker, as graphically shown in Fig 1B.
3. *Route-informed foragers* (*RF*) base their choices on information from long-term memory rather than short-term memory from present experience (Fig 1C). These woodpeckers are knowledgeable about tree availability at coarse spatial scales, such as along their foraging routes or within their home ranges. Individual-based models predict that, based on past foraging experiences, animals should move towards patches exceeding a minimum threshold quality level within patchy landscapes (e.g., [43]). The fitness benefit resulting from the usage of long-term memorized information, however, may decrease as the spatio-temporal complexity in habitat conditions increases in the landscape [3, 24–25, 43]. In this study, we addressed the prevalence of these three foraging behaviors in Magellanic woodpeckers (Fig 1) determining how spatial and temporal variation in tree availability influences tree selection and residence times of woodpeckers.

## Methods

### Ethics statement

This study was conducted in strict accordance with animal ethics approval obtained through Resources of the Ministry of Agriculture of Chile (SAG; resolution N°7415/2014; available at: http://www.sag.cl/sites/default/files/7415_03102014_fauna.pdf). Additional ethics approval was obtained from the Animal Care and Use Committee (IACUC) of the Universidad de Santiago de Chile (resolution N°416-3013/2013). We took all reasonable actions for minimizing the impact on the welfare of the animals during their capture and handling. Birds were trapped using mist nets and handled for the minimum amount of time possible. There was no evidence that VHF and GPS tags affected survival of individuals. Behavioral observations were not considered to cause disturbance. The study was conducted on public lands that did not require specific permissions for access. Magellanic woodpeckers are classified under Least Concern Category of IUCN Redlist of Threatened Species.

### Study landscape

**Fig 2:**
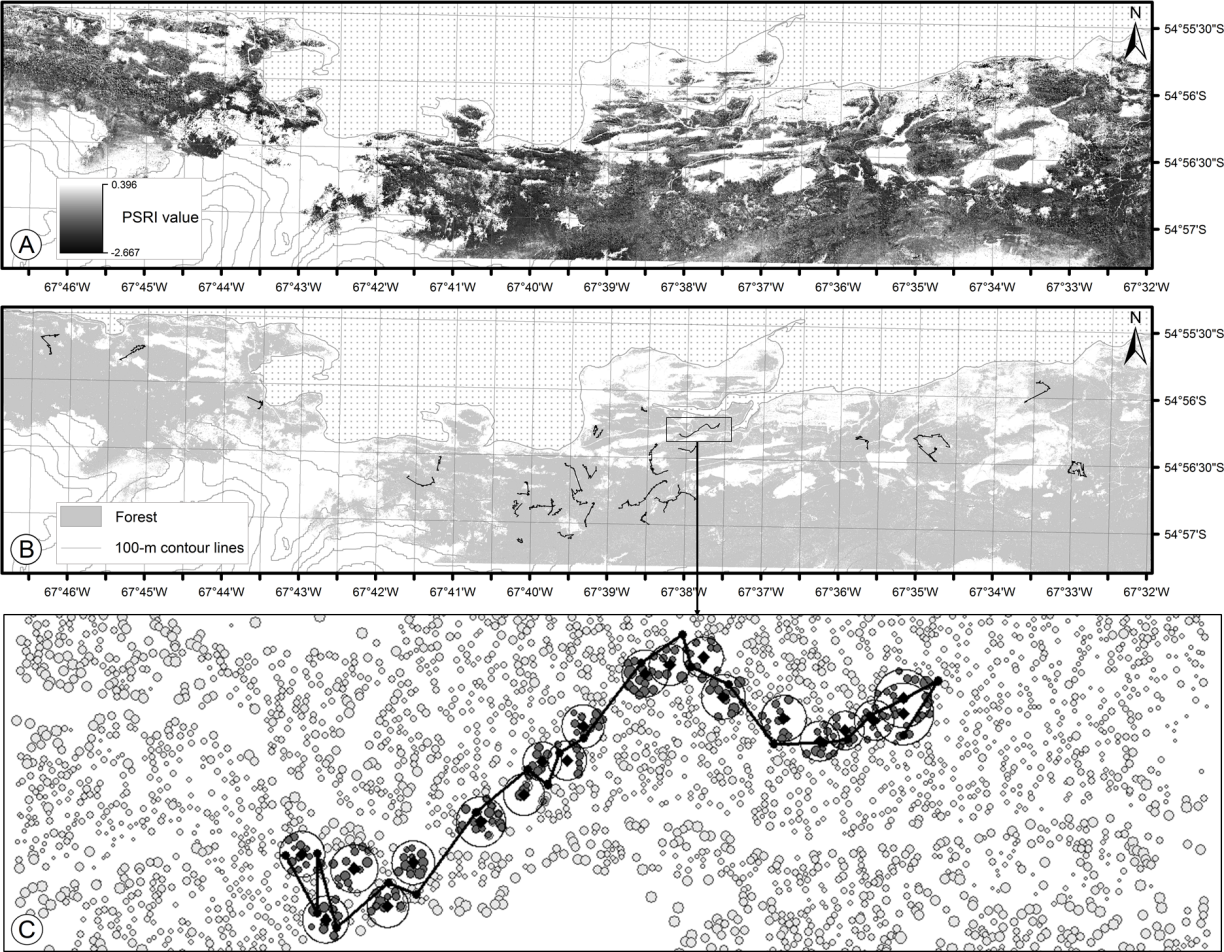
A) Map of the study landscape showing the crowns of individual trees with differences in their Decay Index, as derived from the Plant Senescence Reflectance Index (*PSRI*) of individual trees identified by multiresolution image segmentation. B) Vegetation map distinguishing between forest and non-forest areas as well as including foraging routes from the 14 male Magellanic woodpeckers that were recorded in forest stands within the study landscape. C) Detailed representation of a route (black line) followed by a woodpecker where each tree is represented by a small circle whose size is proportional to its *PRSI* value. The trees available on the route (gray filled small circles) are those contained within the 90% isopleth (large circles denoted by a black line) for a circular distribution generated using Brownian Bridge Movement Model (see Methods section).

This study was conducted in a *ca*. 60 km^2^ area located on Navarino Island (54° 57’ S, 67° 39’ W), Chile (Fig 2). The landscape is dominated by mature southern beech (*Nothofagus* spp.) forest with patches of shrubland, wetlands, peat bogs, and meadows produced by the introduced *Castor canadensis* [53, 54]. Forests in dry and semi-humid areas are comprised of *Nothofagus betuloides* and *N*. *pumilio* with *N*. *antarctica* also occurring in moist and flooded areas. Other evergreen broadleaved trees, such as *Drimys winteri* and *Embothrium coccineum*, occur in low densities [35].

### Woodpecker behavior

We conducted focal observations of male Magellanic woodpeckers during the austral summer (December-March) of 2014 and 2015, corresponding to the post-reproductive season. We focused on adult males because Magellanic woodpeckers forage in pairs, or family groups (n = 3 to 5), with females and juveniles being subordinate to males when foraging [36, 39]. Indeed, dominance exerted by adult males of Magellanic woodpeckers has been observed for males displacing their mates and offspring from tree trunks [39]. By considering adult males only, we avoided introducing variation due to age and sex in our habitat selection analysis. Two trained observers recorded the behavior of the focal woodpecker along its search for larvae through each single trunk and measured each of the used trees along its foraging route (i.e., movement path or bout). Adult male woodpeckers (n = 14), each associated with a different family, were fitted with a VHF transmitter and colored leg band, and then located by using the homing-in method ([55]; see also [35]). Observations were conducted within the known home ranges of focal males, as they have been monitored since 2012 using VHF and more recently (2014-present), GPS technology (Advanced Telemetry Systems, Isanti, MN).

Once located, the focal woodpecker was followed until it flew from sight. We recorded when individuals arrived at and departed from trees used for foraging as well as their general behavior. We distinguished foraging events, such as probing or pecking, from other behaviors that are typical of woodpeckers, such as resting, drumming, vocalizing, and observing (as described in Table A in S1 File; see [31, 36]). When focal woodpeckers were observed using trees, they occasionally disappeared from the observer's visual field. Thus, focal sampling did not provide an accurate estimate of the time spent by woodpeckers in each behavioral activity as well as resulted in the underestimation of behavioral events occurring on short time intervals, such as eating/caching (Table A in S1 File). However, in order to better interpret the behavior of woodpeckers we calculated the percentage of focal observations (number of used trees) in which woodpeckers were observed displaying a particular behavior. To reduce risk of disturbance, birds were observed from distances >20m using 10×42 binoculars. Previous work indicates Magellanic woodpeckers living in Navarino Island are tolerant of humans and seldom changed foraging behavior in the presence of observers [56]. Behavioral sequences (routes) involving fewer than six trees were discarded because they did not provide sufficient data to assess the effect of trees that were available around the previously used trees (see below).

### Tree quality

We determined the “foraging quality” of the trees used by focal woodpeckers and the trees available along each foraging route. Tree quality was estimated by combining field-measurement of georeferenced trees and data extracted from remote sensing imagery, according to the following steps:

1. We characterized trees in terms of their decay stage using categories established by Vergara and Schlatter [32], from healthy trees to dead trees (i.e. rotten with no bark). For each route, we randomly selected trees during behavioral observations (n = 135) and other independent observations from a parallel study (n = 120). Earlier research has established that foraging preferences of Magellanic woodpeckers in southern beech forest are strongly related to decay stage ([32, 34–36]. We also measured other attributes of trees that might affect woodpecker preferences, including the diameter at breast height (measured with a diameter-tape), height (measured with a hypsometer) and species.
2. We estimated the plant senescence reflectance index (*PSRI*) from high-resolution WorldView-2 multispectral imagery. WorldView-2 satellite sensors provide panchromatic and 8-band multispectral imagery at 0.50 m and 2.00 m spatial resolutions, respectively, thus providing appropriate information for the estimation of tree senescence, which is related to woodpecker preference [57]. *PRSI* uses multispectral properties of trees as a proxy of biochemical and biophysical signs of senescence resulting from degradation of chlorophyll and retention of carotenoids concentrations [58]. Larger values of *PSRI* indicate increased senescence or decay-stage of hardwood trees [58, 59].
3. Based on a parallel study, the crowns of individual tree were delimited by subdividing the WorldView-2 scene through multiresolution image segmentation. This approach involved grouping neighboring pixels into regions based on similarity criteria [60]. Image segmentation analysis was based on scale and homogeneity criteria for shape and compactness, respectively, using the software eCognition Developer 64 version 8.7 (Trimble Germany GmbH, Munich, Germany).
4. *PRSI* values were assigned and averaged over the crown of each tree identified in the segmentation process using spatial analysis in ArcMap v. 10.1 (ESRI, Redlands, CA). These scaled values represent our main habitat quality estimate, which we term the “Decay Index” (*DI*).
5. As a preliminary analysis, we used ordinal regression to validate the use of tree-level *PRSI* as a reliable estimator of the decay stage of southern beech trees, and to quantify the relationship. Observed decay stage of trees was treated as an ordinal categorical variable [32]. Preliminary ordinal regressions indicated that the *PSRI* of trees increases significantly as the decay stage of trees increases (*Z* = 7.52; p<0.001), whereas significant pairwise differences in *PSRI* were found among the different decay stages (Table B and Fig A in S1 File). Therefore, we used the tree-level *PSRI* as an index of the decay stage of each tree (see below), hence representing the foraging tree quality for woodpeckers.

### Available trees

We used Brownian bridge movement models (*BBMM*) to identify the trees that were available for woodpeckers each time they moved between two consecutive trees. *BBMM*s produce probability distributions of temporally correlated spatial data under the assumption that animal movement follows a correlated random walk [46]. For each triplet of trees being consecutively used by a woodpecker, *Tr*_*t*_, *Tr*_*t+1*_ and *Tr*_*t*+2_ (where *Tr* is the tree coordinates at a given time), the expected location at the foraging period *t+1*, *μ*(*Tr*_*t*_) results from a linear interpolation between *Tr*_*t*_ and *Tr*_*t*+2_; note that depending on the movement shape *μ*(*Tr*_*t*_) can differ from *Tr*_*t+1*_. The spatial variance in the movement between *Tr*_*t*_ and *Tr*_*t*+2_, *σ*^*2*^_*t*_, indicates the irregularality (sinuousity) of movement between successive trees, being a function of two smoothing parameters:*σ*^*2*^, associated to the speed of the animal, and *δ*, the sampling error (imprecision) in the location of the animal (see *Parameter estimation* section in [46, 50, 61]; see Fig 1). We estimated *σ*^*2*^_*t*_ by using the likelihood function available from the package adehabitatHR (R Development Core Team 2013, [62]) while δ was defined as 3m, corresponding to the average accuracy of the handheld navigator used to georeference each tree used by woodpeckers.

Available trees were defined by fitting circular normal probability distributions centered in the expected locations *μ* (*Tr*_*t*_) from the *BBMM* algorithm and using the spatial variance *σ*^*2*^_*t*_, as described above. Thus, this circular normal function assigns the probability that a tree is locally available for the woodpecker during the period *t* (Fig 1 and Fig 2). We considered as “available” all the trees contained within the 90% isopleth for the circular probability distribution generated using the *BBMM* (see Fig 1 and Fig 2C). Once identified, each available tree *j* was assigned with a *DI* value (*DI*_*j*_), as described in the Tree quality subsection (see above). Thus, the expected *DI* value for all the *n* trees (*j* = 1, 2,…, *n*) that were available during the time period *T* was estimated as:

$$E{\left(DI\right)}_{T}=\frac{1}{n}\sum_{j=1}^{n}DI_{j}$$ (1)

On basis of the three foraging behaviors hypothesized for Magellanic woodpeckers (Fig 1), and using (1), we estimated the Decay Index Ratio (*DIR*) for the *i*^*th*^ available tree along the foraging route *r* and during the current foraging period *t*, as:

1. *Locally-informed foragers* (*LF*): the ratio between the *DI* value of each individual tree available at *t* and the expected *DI* value for all available trees at the current *t* foraging location,
  $$DIR_{i,\Delta t=0}=\frac{DI_{i,t}}{E{\left(DI\right)}_{\Delta t=0}}$$ (2)
  where Δ*t* is the time lag (Δ*t = t–T*, with *t* ≥ *T*) at which the estimation was based (see below). In this case Δ*t* = *0* since *T* = *t*.
2. *Delayed-informed foragers* (*DF*): the ratio between the *DI* value of each individual tree available at *t* and the expected *DI* value for all trees available at the previous Δ*t* foraging locations, such that
  $$DIR_{i,\Delta t\geq 1}=\frac{DI_{i,t}}{E\left(DI_{\Delta t\geq 1}\right)}$$ (3)
  with the time lag 1 ≤ Δ*t* ≤ 4 being constrained by the length of the time series data (see the Woodpecker behavior subsection).
3. *Route-informed foragers* (*RF*): the ratio between the *DI* value of each individual tree and the expected *DI* value for all trees available along the foraging route *r*, such that
  $$DIR_{i,r}=\frac{DI_{i,t}}{E\left(DI_{r}\right)}.$$ (4)
  where *r* = *T*_*max*_−*T*_0_ with *T*_0_ and *T*_*max*_ being, respectively, the initial and final periods at which the woodpecker was tracked along the foraging route *r*. The above estimates of Decay Index Ratio (*DIR*) can be interpreted as the relative quality of a particular micro-habitat feature, which is in essence a habitat suitability index (*HIS*). However, unlike the classical *HIS* approach (e.g., [63, 64]; see also [65]), based on optimum habitat conditions arbitrarily defined by the researcher, *DIR* estimates are based on the animal’s expectancy of habitat quality. Thus, *DIR* behaves as a resource selection index, with *DIR* ≥ 1, representing a tree whose quality is equal, or larger, than the expectations for available trees.

### Tree selection

We used a three-state Bayesian hierarchical model to account for the tree selection pattern of Magellanic woodpeckers. At each foraging period *t*, a tree may be in three different use-states: unused by the woodpecker (*U*), used as a foraging tree (*F*), or used, but not as a foraging site (*O*). The latter state allows distinguishing foraging behavior from other behavioral modes exhibited by Magellanic woodpeckers (Table A in S1 File). The probability of a tree being used in each state during the period *t*, is given by the following expressions:

$$Pr(U_{t})=\left(1-a_{t}\right)+\left[\left(1-f_{t-1}\right)\left(1-W_{t}\right)+f_{t-1}\right]a_{t}$$ (5)

$$Pr(F_{t})=v_{t}\;\left(1-f_{t-1}\right)\;W_{t}a_{t}$$ (6)

$$Pr(O_{t})=\left(1-v_{t}\right)\;\left(1-f_{t-1}\right)\;W_{t}\;a_{t}$$ (7)

where *a*_*t*_ is the probability that the tree is available for the woodpecker during the current period *t*; *f*_*t*−1_ is the probability that the tree was used during the previous period *t*-*1*, with *f*_*t*−1_ = *Pr*(*F*_*t*−1_) + *Pr*(*O*_*t*−1_); *v*_*t*_ is the probability of the tree of being used as a foraging tree during the period *t*; *W*_*t*_ is the probability that the tree is selected by the woodpecker during period *t*. Because we used a discrete rather than continuous metric, we considered *a*_*t*_ to be a dummy variable taking value 1 if the tree was within the 90% isopleth circular area describing tree availability during period *t*, and otherwise 0 (see Available trees subsection). Given that Magellanic woodpeckers do not use the same trees over successive periods, the probability of a tree being used during period *t*, *Pr*(*F*_*t*_) + *Pr*(*O*_*t*_), was directly proportional to its probability of not being used during the previous period (i.e. to 1—*f*_*t*−1_). The probability of a woodpecker exhibiting a foraging behavior (*v*_*t*_) was modeled as a uniformly distributed latent variable, varying from 0 to 1 during each foraging period. The selection probability of the *i*^*th*^ tree along the route *r*, *W*_*i*,*r*_, as stated in (5), (6), and (7), was specified through the following logit-function:

$$logit(W_{i,r})=\beta_{0}+\beta_{1}\;DIR_{i}+sp_{i}+\gamma +\rho N_{r}$$ (8)

where *β*_0_ is an intercept, *β*_1_ is the coefficient associated with the Decay Index Ratio (*DIR*) of the *i*^*th*^ tree, measured at different time periods and spatial scales (see model selection below), *sp*_*i*_ is a factor for the tree species whose levels were: *N*. *pumilio*, *N*. *antarctica*, *N*. *betuloides* and other evergreen species. Parameter *ρ* represents the route-level random effect of the family size (*N*), whereas parameters *γ* are the random effect parameters for each individual woodpecker (see below for prior distributions).

We built models representing the different foraging behaviors of woodpeckers (*DF*, *LF* and *RF*, as explained in Fig 1). The positive significant Pearson's correlation coefficients (0.67≥ r ≤ 0.84; p<0.01) between the Decay Index Ratios (DIR) measured at different time lags (Δt = 0, 1,.., 4) precluded us from including these covariates in the same models, and hence to directly to compare the hypothesized foraging strategies (see pairwise correlations in Fig B in S1 File). In addition, values of *DIR* measured at different lags involved different time series length. Despite difficulties arising from collinearity and uneven sample sizes, we used a model-selection procedure intended to compare the goodness of fit of models representing woodpeckers behaving as *RF* with models consistent with *LF* and *DF*, with the latter ones involving *DIR* measured at different lags (see the following).

In the first model selection step, estimated *DIR* values at the route-level (*DIR*_*r*_) were regressed onto *DIR* values measured at different time lags (*DIR*_Δ*t*≥0_), with the standardized residuals extracted from these regressions being used as corrected measures of *DIR*_Δ*t*≥0_ (see correlation matrices in Fig B in S1 File). Since the effect of *DIR*_*r*_ may be influenced by the number of trees (*n*_*r*_) sequentially used by a woodpecker along a route *r*, we developed a separate model with the interaction between these two covariates and retained this interaction in posterior models only if it was significant (see below). Second, for each lag (Δ*t*) we specified a set of candidate models resulting from the combinations of *DIR*_*r*_, the corrected (residual) values of *DIR*_Δ*t*_ (when *DIR*_*r*_ was included in the model) and the uncorrected values of *DIR*_Δ*t*_ (when *DIR*_*r*_ was not included). Thus, this set of models comprised three models including either the terms *DIR*_Δ*t*_, *DIR*_*r*_ or *DIR*_Δ*t*_ + *DIR*_*r*_ as fixed-effects whereas the random effects described above were included in all models. An additional null model (without fixed-effects) was added to the set of candidate models and used it as a goodness-of-fit test for these effects. Third, we compared the support of these four models by using the Deviance Information Criteria (*DIC*; [66]) and differences in *DIC* (Δ*DIC*) were used to interpret the strength of evidence for each model. Models with Δ*DIC<2* were considered to be supported by the data. The importance of each fixed effect coefficient was evaluated with Bayesian Credible Intervals (*BCI*) estimated from posterior distribution of parameters. The 95% BCIs that did not overlap zero were considered as being significant. We also estimated the inclusion probability (*P*) of coefficients, which were interpreted as the probability a covariate of being included in the best-supported models [66]. We assumed prior probabilities of *P* were Bernoulli distributed with parameter of 0.5.

The state of each tree (*y*) was modeled with a categorical likelihood function *y* ∽ *cat*[*p*(*U*),*p*(*F*),*p*(*O*)]. We used vague non-informative prior distributions for all model parameters. Parameters *γ*_*r*_ were assumed to be Gaussian distributed associated to each individual woodpecker. Parameter distributions were based on three Markov Chain Monte Carlo (MCMC) samples, each with 20,000 iterations, discarding the first 10,000 iterations and thinning by 3. Model convergence was assessed visually and diagnosed using the Gelman–Rubin test (i.e., Potential Scale Reduction Factor, PSRF) which considers the variance between MCMC chains [67]. Models were run using WinBUGS v. 1.4 via the R-interface R2WinBUGS [68].

### Tree residence time

We used Cox proportional hazard models to evaluate the length of time woodpeckers foraged in trees of different quality [69]. We considered only trees where woodpeckers were observed foraging (termed “foraging trees; see Table A in S1 File). Cox models were used to analyze the probability per unit time that a woodpecker would leave a tree, with this stochastic decision rule being influenced by ecological factors (see below). The rate of leaving the *i*^*th*^ tree at time period *T*, *h*(*T*) (i.e., the observed hazard rate), is the product of a baseline hazard, *h*_0_(*T*), and an exponential function combining the effects of a set of explanatory variables. Thus, the leaving tendency in the *i*^*th*^ tree used in the route *r* is given by:

$$h\left(T_{i,r}\right)=h_{0}\left(T_{i,r}\right)\;exp\left[\beta_{1}DIR_{i}+\beta_{2}DIS_{i}+\beta_{3}DBH_{i}+\beta_{4}HT_{i}+sp_{i}+\rho N_{r}\right]$$ (9)

where the parameters *β*_1_, *sp*_*i*_ and *ρ* are the same as in (8), while *β*_2_, *β*_3_, *β*_4_ are the coefficients of the travel distance (*DIS*, in m), diameter at breast height (*DBH*, in cm) and height (*HT*, in m) of the *i*^*th*^ tree, respectively. *DIS* was included to be consistent with the predictions of the Marginal Value Theorem (MVT, [2]). According to the MVT, a woodpecker should spend more time in a given tree as the distance or travel time to other trees increase [70].

We assumed a prior gamma process for the baseline hazard, *h*_0_(*T*) [71]. Bayesian model specifications and parameter estimation were similar to those described above for the tree selection models, with models being selected through an information-theoretical approach based on the *DIC*. First, we defined a set of candidate models including different combinations of the Decay Index Ratio *DIR*, *sp*, *DIS*, *DBH* and *TH* in (9), whereas the random effect *ρ* was retained in all models. However, we only included into the set of candidate models those *DIR* estimates whose effects were supported by tree selection models (as explained in the previous analysis). This simplification in the number of covariates not only reduced the number of candidate models (from 2^10^ possible models), but also restricted the analysis to determine how foraging preferences were related to tree attributes known to affect residence time. The relative contribution of each covariate affecting tree residence time was evaluated by examining the BIC of regression coefficients (*β*) and by exponentially transforming these coefficients, *exp*(*β*), indicating the hazard changes with a unit increment of the covariate. If *exp*(*β*) > 1, an increase in the covariate results in the woodpecker leaving the tree earlier, while if *exp*(*β*) < 1, the woodpecker should stay longer at that tree [69].

## Results

We obtained data on 39 foraging routes used by 14 adult male Magellanic woodpeckers (Table C in S1 File). Routes included between six and 33 trees sequentially used by woodpecker individuals. On average, the number of trees that BBMMs identified as available at each tree location ranged between 7 and 16 trees (Table A in S1 File). Our analyses included 4714 available trees, of which 424 were used by woodpeckers and 4290 trees were not used (tree data are available at Table A in S1 File). Of the used trees, in 397 (94%) of them woodpeckers were recorded displaying some foraging behavior (Table A in S1 File). Woodpeckers elicited a sequence of behaviors once they arrived to foraging trees (see the behavior's prevalence in Table A in S1 File). First, individuals started walking along the trunk, or branches, often switching to a short probing behavior. When birds encountered rotten wood, they pecked more intensively through a Probing/Pecking behavior (Table A in S1 File). Some individuals continued to peck and displayed an intensive drill (Tapping) until reaching the feeding item in the core wood (Eating/Catching; Table A in S1 File). Walking (81.6%), Short probing (76.2%) and Probing/pecking (51.0%) were the most prevalent behaviors observed in focal woodpeckers (Table A in S1 File).

### Tree selection

**Fig 3:**
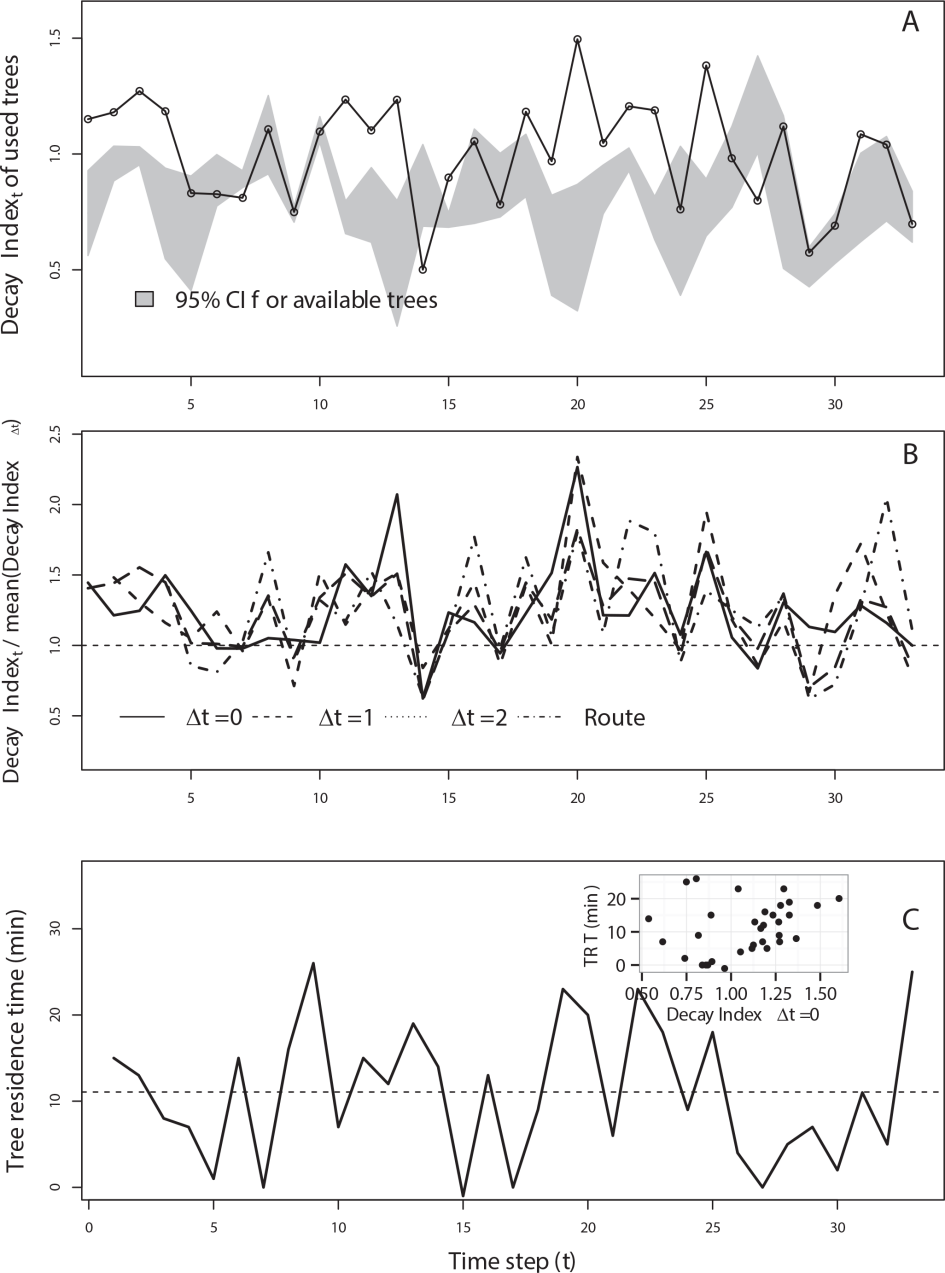
Sequence of foraging decisions made by a male Magellanic woodpecker. A) Decay Index (*DI*) of the trees used by the woodpecker, quantified through their transformed values of plant senescence reflectance index (PSRI). These values are compared with the Confidence Intervals (*CI*) of the *DI* values for all trees available at the current tree's location. B) Decay Index Ratio (*DIR*) of used trees estimated as the ratio between the *DI* of the tree and the mean *DI* for all trees available over different periods and spatial scales. *DIR* were estimated at the route-level (*DIR*_*r*_) and at different lags (*DIR*_Δ*t*_, with 0≤ Δ*t* ≤4). C) The residence time spent by the woodpecker in each used tree (*TRT*). The inserted scatter plot shows the positive association observed between *DIR* and *TRT*.

Magellanic woodpeckers selected trees with a higher decay stage than available trees, as measured with the Decay Index (Fig C in S1 File; Fig 3A). Consistent with this woodpecker’s selectivity pattern, the Decay Index Ratio (*DIR*) relative to the trees available at the route-level and over different lags (0 ≤ Δ*t* ≤ 4) tended to be larger than unity (i.e., *DIR*_*r*_ ∧ *DIR*_Δ*t*≥0_ > 1; Fig 3B). However, there were quantitative differences among these *DIR* estimates, as being inferred from the supported models described below (see Fig 3B).

The best-supported models comparing the importance of tree quality at local (*DIR*_Δ*t* = 0_) and route (*DIR*_*r*_) levels indicated that Magellanic woodpeckers behaved as locally-informed (*LF*) rather than route-informed foragers (*RF*; Table 1). However, when compared with the quality relative to trees available at previous periods (1≤ Δ*t* ≤ 4), we found support (Δ*DIC* ≤ 2) only for the models that included *DIR*_*r*_ (Table 1). No models that included both the effects of standardized residuals and uncorrected values of *DIR* estimated with lags ≥ 1 (*DIR*_*i*,Δ*t*≥1_) were supported by data (Table 1).

**Table 1:** Models explaining the tree selection probability of Magellanic woodpeckers as a function of the Decay Index Ratio (*DIR*), which represents the quality of each tree relative to the mean decay stage of all trees available at different time lags (Δt = 0, 1, 2, 3 and 4) and spatial-scales (route vs. local-scale), as explained in the main text. Model selection was carried out for each period independently, with models being ranked according their *DIC* values. Models with Δ*DIC* <2 were considered to be supported. The model with intercept-only (*β*_0_) was considered as a 'baseline' or 'null' model.

Model		Deviance				DIC	ΔDIC
		mean	SD	2.5%	97.5%		
Tree quality relative to trees available at Δt = 0 (N = 4714)							
	*DIR*_Δt = 0_	2465.4	28.9	2408.9	2524.9	2634.1	0.0
	*DIR*_r_ + *DIR*_Δt = 0_	2468.7	28.2	2410.4	2520.1	2637.6	3.5
	*DIR*_r_	2470.0	29.2	2409.4	2521.3	2639.0	4.9
	*Null*	2711.5	31.8	2408.0	2521.7	2897.1	262.9
Tree quality relative to trees available at Δt = 1 (N = 4293)							
	*DIR*_r_	2234.4	27.2	2406.9	2520.6	2387.2	0.0
	*DIR*_r_ + *DIR*_Δt = 1_	2236.6	28.0	2408.3	2525.1	2389.6	2.4
	*DIR*_Δt = 1_	2240.9	28.2	2406.6	2522.2	2394.2	7.0
	*Null*	2513.9	29.9	2409.9	2523.5	2685.9	298.7
Tree quality relative to trees available at Δt = 2 (N = 3872)							
	*DIR*_r_	2015.5	26.1	2408.3	2522.4	2153.3	0.0
	*DIR*_r_ + *DIR*_Δt = 2_	2018.0	26.8	2407.9	2520.7	2155.9	2.6
	*DIR*_Δt = 2_	2022.4	26.1	2408.0	2523.2	2160.7	7.4
	*Null*	2253.7	30.6	2408.5	2522.9	2407.9	254.6
Tree quality relative to trees available at Δt = 3 (N = 3451)							
	*DIR*_r_	1802.7	24.0	2410.5	2522.9	1925.9	0.0
	*DIR*_r_ + *DIR*_Δt = 3_	1805.5	25.2	2408.7	2524.3	1928.9	3.0
	*DIR*_Δt = 3_	1811.8	25.1	2408.2	2523.2	1935.6	9.7
	*Null*	2303.5	27.4	2410.5	2523.6	2461.0	535.2
Tree quality relative to trees available at Δt = 4 (N = 3030)							
	*DIR*_r_	1586.8	22.0	2408.8	2520.2	1695.2	0.0
	*DIR*_r_ + *DIR*_Δt = 4_	1589.7	23.3	2409.2	2521.8	1698.3	3.1
	*DIR*_Δt = 4_	1600.4	23.6	2409.0	2523.8	1709.7	14.6
	*Null*	1813.7	23.5	2405.4	2523.3	1937.6	242.5

**Fig 4:**
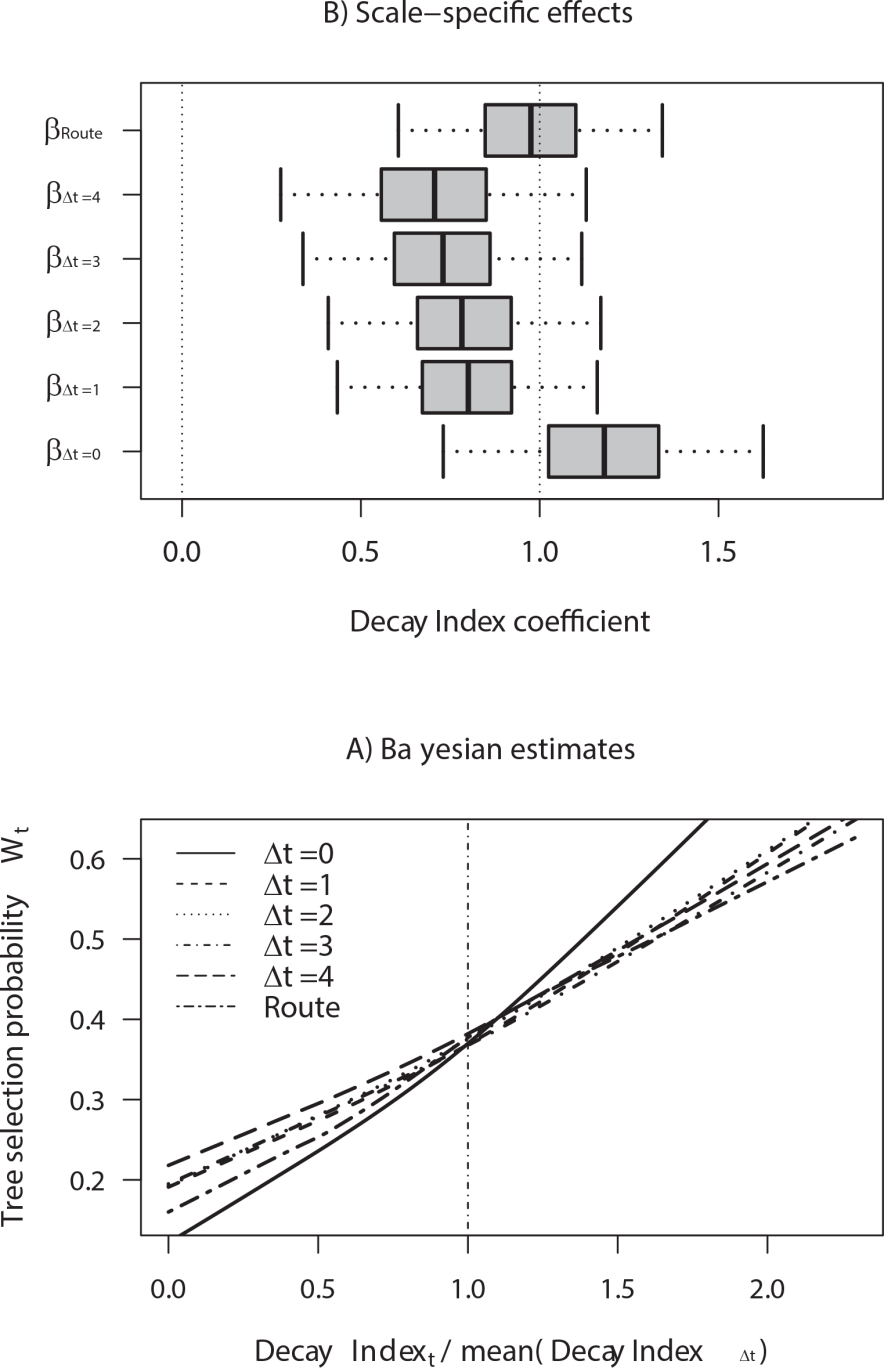
Posterior estimates derived from Bayesian tree-use state models accounting for the trees selected by Magellanic woodpeckers. A) Boxplot showing the fixed-effect coefficients (*β*) associated to *DIR* estimated at different time lags (Δt = 0, 1, 2, 3 and 4) and spatial-scales (route vs. local-scale; see details in Table 2). B) The increase in the tree selection probability (*W*) as a function of *DIR* estimated over different periods and spatial scales

Model coefficients indicated that *DIR* estimated over different periods and spatial scales were significantly related to the probability a tree was selected by a woodpecker (*W*), as shown by the 95% Bayesian Credible Intervals (BCI) of the coefficients associated with these covariates (Table 2; Fig 4A). With the exception of the coefficient associated with *DIR*_Δ*t* = 4_, the inclusion probabilities (*P*) of *DIR* coefficients were ≥ 0.95, indicating high levels of confidence (Table 2). Tree selection probability (*W*) increased with the tree quality (*DIR*), but such an increase in *W* was more pronounced when the quality was relative to the current local-level, as shown by the steeper logit slope coefficient (*β* > 1) for *DIR*_Δ*t* = 0_, when compared with the slopes for *DIR*_*r*_ and *DIR*_Δ*t*≥1_ (*β* < 1; Fig 4A and 4B). Coefficients associated with the "standardized residuals" of *DIR* (obtained from *DIR*_Δ*t*_ ∼ *DIR*_*r*_ regressions), however, were not significant and had *P* < 0.1 (with the exception of the coefficient associated with *DIR*_Δ*t* = 0_ for which *P* = 0.6; Table 2). The coefficient (*η*) for the interaction between *DIR*_*r*_ and the route length (*n*_*r*_) was not significant (Table 2), whereas model coefficients supported neither the effects of the tree species nor the effect of family size on the tree selection by woodpeckers (Table 2).

**Table 2:** Coefficients of the tree selection models described Table 1 and Eq 4, inclu0064ing standard deviations (SD), 95% Bayesian Credible Interval (BCI) and inclusion probability (P, with larger values indicating greater confidence) and the Potential Scale Reduction Factor (PSRF) for convergence diagnostic (with values close to 1 indicating approximate convergence). Coefficients include the effects of the Decay Index Ratio (DIR), estimated at different time lags (Δt = 0, 1, 2, 3 and 4) and spatial-scales (route vs. local-scale). Coefficients associated with the "standardized residuals" of DIR refer to the residuals of the corresponding covariate obtained after regressing on the covariate (see model variables in Eqs 2,3 and 4 and the main text).

Covariate	Untransformed variables						Standardized residuals				
	mean	SD	BCI		P	PSRF	mean	SD	BCI		P
			2.50%	97.50%					2.50%	97.50%	
*DIR*_r_	0.96	0.18	0.61	1.31	1.000	1.001	-	-	-	-	-
*DIR*_Δt = 0_	1.17	0.19	0.8	1.54	1.000	1.001	0.77	1.98	-2.47	4.15	0.601
*DIR*_Δt = 1_	0.78	0.16	0.47	1.1	1.000	1.001	0.10	5.24	-9.25	9.31	0.049
*DIR*_Δt = 2_	0.76	0.17	0.44	1.11	1.000	1.002	0.07	5.28	-9.31	9.31	0.044
*DIR*_Δt = 3_	0.81	0.17	0.51	1.18	0.977	1.002	-0.01	5.22	-9.3	9.15	0.052
*DIR*_Δt = 4_	0.68	0.67	0.31	1.09	0.886	1.002	0.03	5.25	-9.28	9.24	0.051
*N betuloides*	-2.05	3.01	-7.17	3.09	0.025	1.192	-	-	-	-	-
*N*. *antarctica*	-2.13	3.03	-7.34	3.12	0.033	1.191	-	-	-	-	-
Other tree spp.	-0.56	4.9	-8.97	8.19	0.015	1.286	-	-	-	-	-
Foraging mode (*v*)	0.94	0.03	0.89	1	-	-	-	-	-	-	-
Family size (*ρ*)	-0.02	0.19	-0.38	0.35	0.01	1.001	-	-	-	-	-
Route length (*η*)	0.05	0.12	-0.2	0.29	0.077	1.207	-	-	-	-	-

In Table 2, coefficients for the foraging mode probability (*v*_i_) were averaged on all trees.

### Tree residence time

Woodpeckers remained 8.25 ± 0.58 min (mean ± SE) on foraging trees, with residence times ranging between 0.42 and 64.33 min (Fig D in S1 File). Cox’s proportional hazards models showed that Magellanic woodpeckers adjust their tree residence times based on the tree Decay Index Ratio of the trees available along the route (*DIR*_*r*_), as shown for the best supported (Δ*DIC* ≤ 2) model in Table 3. We did not find support for the Cox model including the effect of the tree quality relative to the current local-level (*DIR*_Δ*t* = 0_, Table 3). Hence Magellanic woodpeckers behaved as route-informed foragers (*RF*) when adjusting their times on each tree. In addition, the tree residence time was affected by other attributes of selected trees, including diameter at breast height (*DBH*), tree species (*sp*) and travel distance between trees (*DIS*; Table 3).

**Table 3:** Results from the Cox proportional hazards models analyzing the association between the tree residence time of foraging woodpeckers and attributes of individual trees. Fixed-effect variables include: 1) the Decay Index Ratio (*DIR*): the quality of each tree relative to the mean decay stage of all trees available at the local scale (*DIR*_Δ*t* = 0_) or along the route (*DIR*_*r*_, see the main text); 2) *DIS*: travel distance between trees; 3) *DBH*: diameter at breast height of the tree; 4) *TH*: height of the tree; and 5) *Sp*: tree species. Models were ordered according to their DIC weights and ΔDIC, with ΔDIC <2 indicating that the model is supported by data.

Model*	Deviance				DIC	ΔDIC	Weight
	mean	SD	2.5%	97.5%			
*DIR*_r_ + *DIS + DBH + Sp*	1884.6	8.9	1869.0	1904.0	1909.7	**0.0**	0.942
*DIR*_r_ + *DIS* + *DBH*	1894.4	8.6	1879.0	1912.0	1916.2	6.5	0.037
*DIR*_Δt = 0_ + *DIS + DBH + TH + Sp*	1892.9	9.1	1876.0	1912.0	1918.8	9.2	0.010
*DIR*_Δt = 0_ + *DIS* + *DBH + Sp*	1893.8	9.2	1877.0	1912.0	1919.0	9.4	0.009
*DIR*_Δt = 0_ + *DIS + DBH + TH*	1899.3	8.9	1883.0	1917.0	1921.9	12.2	0.002
*DIR*_Δt = 0_ + *DIS* + *DBH*	1900.8	9.1	1884.0	1919.0	1923.0	13.3	0.001
*DIS*	1942.7	20.0	1905.0	1985.0	1962.8	53.2	0.000
*DIR*_Δt = 0_ + *DIR*_r_ + *DIS*	1953.0	12.9	1927.0	1978.0	1975.0	65.3	0.000
*DIR*_r_ + *DIS*	1961.1	14.8	1935.0	1990.0	1981.9	72.2	0.000
*DIR*_Δt = 0_	1959.7	14.1	1933.0	1990.0	1983.5	73.8	0.000
*DIR*_r_	1960.1	14.1	1933.0	1989.0	1984.0	74.3	0.000
*DIR*_Δt = 0_ + *DIS*	1997.2	15.1	1968.0	2028.0	2018.1	108.5	0.000
*DIR*_Δt = 0_ + *DIS + Sp*	2002.2	16.9	1970.0	2036.0	2022.0	112.3	0.000
*DIR*_r_ + *DIS + Sp*	2010.2	17.5	1977.0	2044.0	2029.9	120.2	0.000

**Fig 5:**
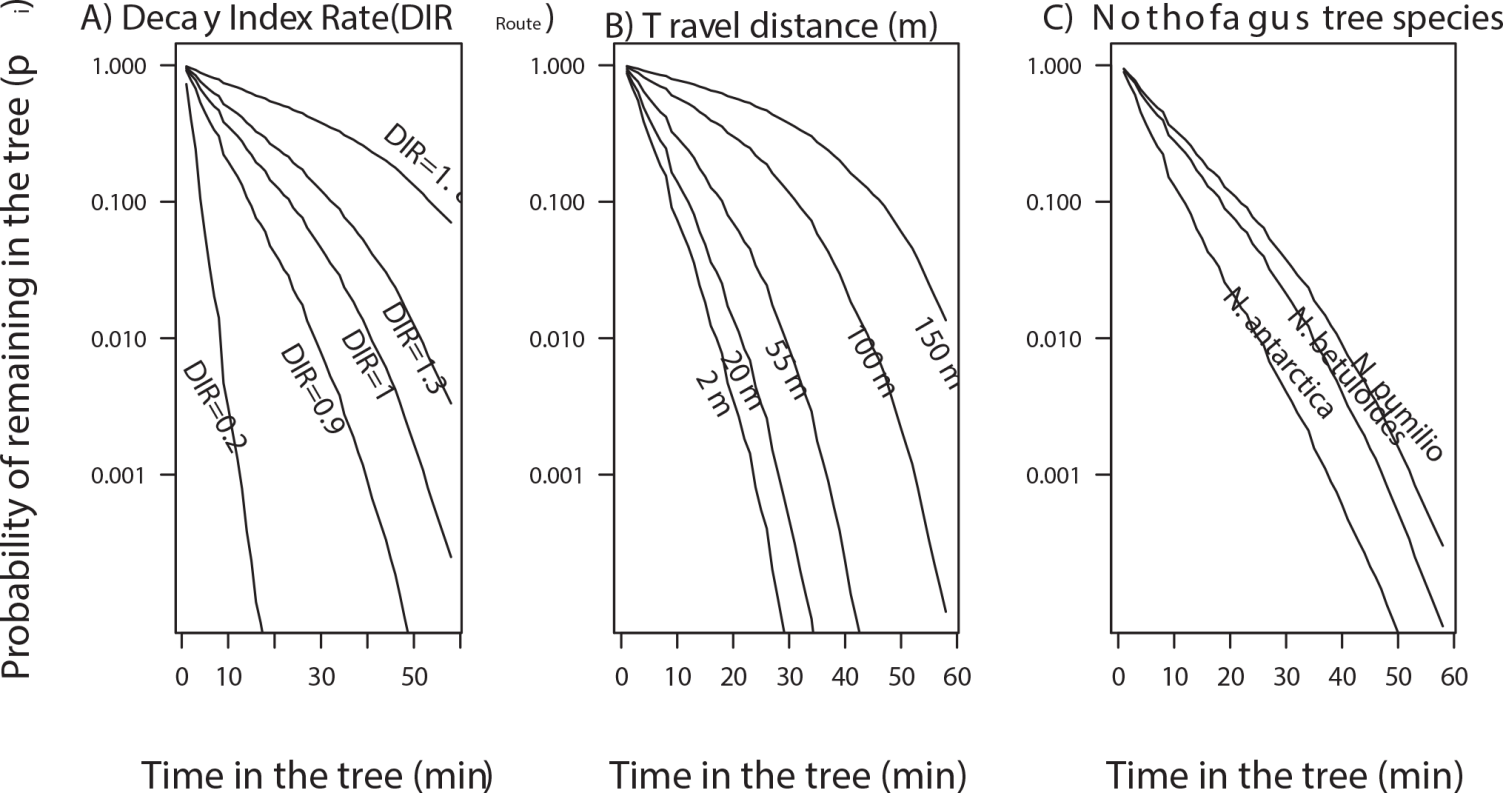
Probability that a Magellanic woodpecker will remain in a tree as its time in the tree increases, as estimated from Cox proportional hazards models described in Tables 3 and 4. Curves exhibiting a rapid decay indicate that the tree leaving tendency is increased, which is the case of: 1) Trees with low values of Decay Index Ratio relative to the trees available along the route (e.g., for trees with *DIR*_*r*_ <1); 2) Travel distances < 20 m; and 3) Trees of *Nothofagus antarctica*.

Coefficients of the Cox models indicated that woodpeckers spend relatively more time on trees of higher quality (*DIR*_*r*_), with larger diameters, and at increasing distances to other trees as indicated by the significant negative values for the coefficients and their respective hazard ratios, which were < 1 (Table 4). Conversely, woodpeckers left *Nothofagus betuloides* or *N*. *antarctica* earlier than when foraging on *N*. *pumilio* and other evergreen trees (e.g., *Drimys winteri* and *Embothrium coccineum*; Table 4). Curves for the time-dependent probability of woodpeckers to remain in the tree revealed an increased tree-leaving tendency in trees with low *DIR*_*r*_ values (Fig 5). In fact, a woodpecker using a tree with a decay state equal to the expected for the trees available along the route (i.e., trees with *DIR*_*r*_ = 1) should spend only 3.4 min in the tree, whereas a woodpecker should spend 14.5 min in a tree belonging to the upper quartile (> 75%; *DIR*_*r*_ = 1.8) the (Fig 5). Reduced travel distances, especially less than 20 m (the lowest quartile of travel distances), resulted in shorter residence times (Fig 5; Fig E in S1 File). In addition, the probability of remaining in a *N*. *pumilio* tree was ca. 2 and 9 times higher than for *N*. *betuloides* and *N*. *antarctica*, respectively (Fig 5).

**Table 4:** Estimated coefficients of Cox proportional hazards models for the tree residence time of woodpeckers, including standard deviations (SD), 95% Bayesian Credible Intervals (CI) and hazard ratios (HR). Model covariates are described in Table 3 and *Eq* 5.

Covariate	mean	SD	BCI		HR
			2.5%	97.5%	
*DIR*_Δt = 0_	-2.33	0.138	-2.60	-2.05	0.10
*DIR*_r_	-2.47	0.130	-2.72	-2.21	0.08
*DIS*	-0.02	0.002	-0.02	-0.01	0.98
*DBH*	-0.03	0.003	-0.04	-0.03	0.97
*HT*	-0.01	0.003	-0.01	0.00	1.00
*N betuloide*	0.31	0.125	0.08	0.56	1.37
*N*. *antarctica*	0.81	0.268	0.26	1.30	2.24
Other tree spp.	-0.40	0.257	-0.93	0.08	0.67
Family size (*ρ*)	0.40	1.083	-1.70	2.45	1.50

## Discussion

Results of this study are consistent with theoretical models of habitat selection and optimal foraging, which assume that animals living in patchy habitat adjust their preferences for, and the time spent in each foraging patch to the quality expected for the patches that are “available” [1–3, 72, 73]. That said, our study is novel because we integrate theoretical and empirical methods to explicitly test the spatio-temporal scales at which animals perceive habitat availability in real landscapes, which is an approach seldom applied (e.g., [74]). The best-supported models suggest that foraging decisions of Magellanic woodpeckers are based on information gathered across multiple spatio-temporal scales. Our observations also reveal that woodpeckers use trees as predominantly foraging substrates when moving along routes. When selecting an individual tree, woodpeckers based their preference on information in the immediate vicinity, as estimated by *DIR*_Δ*t* = 0_. In contrast, when deciding how long to forage on a particular tree, woodpeckers used information compiled across broader spatial scales (route level), as estimated by *DIR*_*r*_. Our data suggest that woodpeckers do not behave as delayed-informed foragers and instead use both long-term and updated information to guide foraging decisions. One potential cost of the delayed-information strategy is that woodpeckers may be relatively inefficient in selecting trees suitable for foraging and adjusting residence times. The scale-dependent perception of habitat by wild animals has been addressed by previous studies of hierarchical habitat selection [30, 75, 76] or movement patterns [77–79]. Collectively, this body of work shows that foraging decisions reflect an interaction between habitat characteristics and organism-level processes, such as the animal’s internal state, motion capacity and navigational capacity [13].

Our study shows that Magellanic woodpeckers acquired information at increasing spatial scales: the within-tree, clusters of neighboring trees, and trees distributed across the home range. While foraging on a particular tree, our focal species, like many woodpeckers, used a series of exploratory behaviors to assess the presence of wood-boring larvae into the trunk or branches (e.g., probing or pecking; Table A in S1 File). However, further studies are necessary to estimate the rate at which woodpeckers succeed in capturing larvae. Drawing upon experiences at previously visited trees may promote foraging efficiency when food items are cryptic or remaining hidden into the trees, such as wood-boring larvae [80, 81]. The pattern of tree residence time exhibited by Magellanic woodpeckers is consistent with a Bayesian foraging behavior, with woodpeckers adjusting times by combining prior information (e.g., collected at the route level) with that resulting from the instantaneous harvest rate in the tree (e.g., [41, 82]), including tree-level attributes such as the trunk's diameter [52]. In particular, the use of tree information across broad spatial scales (*DIR*_*r*_) involved knowledge acquired as long-term memory. Home-range data collected for the 14 woodpeckers of this study indicated that territories of focal families remained stable across seasons (reproductive and post-reproductive) and years (2012–2015). Consequently, these woodpeckers should have accumulated information on the trees that were used repeatedly across their home ranges, which is expected for animals with stationary home ranges that are knowledgeable about the spatio-temporal distribution of habitat suitability [27, 30, 43]. Woodpeckers using long-term spatial memory, however, could avoid recently visited trees, as observed in woodpeckers moving along routes. Woodpeckers might use long-term memory to adjust feeding schedules based on the renewal rate of resources at trees, as suggested by theoretical models [27, 43]. The lack of memory-based decision-making might weaken woodpecker preferences for decayed trees since the availability of larvae in the intensively used trees could fall below the levels of quality perceived by woodpeckers.

When woodpeckers selected individual trees, these decisions were based on the clusters of trees around the most recently visited trees, as identified using the BBMM approach (Fig 1, Fig 2C; see [51]). The relatively short distances from the woodpecker to trees identified as available by BBMMs (usually less than 15 m) suggest that woodpeckers use visual cues from neighboring trees, information that is internally processed and used as a proxy of the tree quality [83, 84]. Indeed, the perceptual range of an animal determines its movement through the landscape and the minimum spatial scale (“functional grain”) at which it recognizes spatial heterogeneity [85, 86]. Although further study is required, we suggest that Magellanic woodpeckers gather visual information from the nearby surrounding trees due to these trees being located within their perceptual range.

The sequential behavior exhibited by foraging woodpeckers suggests that the woodpeckers staying longer in trees are more likely to extract wood-boring larvae. Thus, results are consistent with optimal foraging theory, with residence times being longer in trees whose quality is higher than that of the trees available in the home-range [3, 4]. Indeed, the best-supported tree selection and Cox models indicated that Magellanic woodpeckers were knowledgeable about the quality of trees available at different spatial scales and, hence, behaved as locally-informed foragers (*LF*) and route-informed foragers (*RF*) when they selected foraging trees and adjusted their time in these trees, respectively. We suggest that the sequential switching between these two behavioral modes (*LF* and *RF*) involved a general foraging strategy based on the decay stage of trees that woodpeckers perceived and memorized along foraging routes. The movement pattern exhibited by foraging woodpeckers, which involved short travel distances between the sequentially visited trees (28.7 ± 0.82 m; S5 Fig), resembles the behavior of some animals that repeat the same foraging routes through the “best quality” sites, usually referred to a “trapline foraging” [85]. Trapline foraging has been described not only for nectarivore insects and hummingbirds (e.g., [87]), but also for insectivore birds (e.g., [88]) and mammals. Although it remains to be studied whether Magellanic woodpeckers can maximize feeding rates by revisiting the same routes across their home ranges (as expected for trapliners), our results suggest that woodpeckers adjust memorized information with short-term information available within their perceptual ranges. Short- and long-term information sources would differ in their function, with the former one being used to orient movement towards the best quality trees, whereas the latter is used to establish an a priori estimate of tree quality. Such a mixed foraging strategy must be understood as emerging from the movement pattern of foraging woodpeckers (characterized by short-distance movements), the spatio-temporal variation in the quality of trees within home-ranges as well as the cognitive and perceptual capacities of woodpeckers. We suggest that Magellanic woodpeckers are imperfectly informed about the spatial location of individual trees, perhaps due, in part, to the spatio-temporal complexity of the landscape and large home ranges (typically covering about 100 ha) that can include high numbers of trees (ca. 10,000 trees). Woodpeckers also may be limited by lack of information about the resource renewal schedule in each tree within their home ranges (dependent of the life history of the wood-boring larvae). Uncertainty resulting from this spatio-temporal variation in habitat quality could lead Magellanic woodpeckers to use simple heuristic rules when foraging, as described above, rather than using long-term spatial memory to memorize the spatial location of each individual tree. That said, Magellanic woodpeckers are likely informed about the high-quality patches (or forest stands) within their home ranges [43], thus orienting movements towards sites of greater resource concentration [9]. Thus, our work provides supportive evidence that Magellanic woodpeckers display foraging and movement strategies that integrate the information on habitat quality collected at different spatial scales.

## Supporting Information

S1 File **Table A.** Main behaviors elicited by Magellanic woodpeckers according to Short (1970) and classified into two behavioral states (see main text). **Table B.** Coefficients, standard errors and p-values from an ordinal regression model evaluating the contribution of the Plant Senescence Reflectance Index (*PSRI*, see text) to the observed decay state of the trees used by Magellanic woodpeckers. **Fig A.** Differences in the Plant Senescence Reflectance Index (PSRI) among the trees sampled during behavioral observations. **Fig B.** Pearson's correlation coefficients between estimates of the tree Decay Index Ratio (*DIR*). **Table C.** Summary of foraging routes followed by Magellanic woodpeckers recorded within forest patches. **Fig C.** Distribution of tree selection probability (*W*) for the trees used and not used by Magellanic woodpeckers. **Fig D.** Distribution of residence times (min) for the trees used by Magellanic woodpeckers. **Fig E.** Distribution of travel distances (m) between trees used by Magellanic woodpeckers.
(DOCX)

S2 File Tree data containing the following variables: PSRI = untransformed values of PRSI for all trees used by woodpeckers; Tree.stage = the foraging stage of each tree (unused trees: 0, used for foraging: 1, used for other behaviour: 2); Time = time step along the route; Route = the number of the route. (XLSX)
