# Supplementary material for: Foraging Behaviour in Magellanic Woodpeckers Is Consistent with a Multi-Scale Assessment of Tree Quality
Source: PLoS One. 2016 Jul 14;11(7):e0159096. doi: 10.1371/journal.pone.0159096 (PMC4945014; doi:10.1371/journal.pone.0159096)
Supplement: S1 File — Table A. Main behaviors elicited by Magellanic woodpeckers according to Short (1970) and classified into two behavioral states (see main text). Table B. Coefficients, standard errors and p-values from an ordinal regression model evaluating the contribution of the Plant Senescence Reflectance Index (PSRI, see text) to the observed decay state of the trees used by Magellanic woodpeckers. Fig A. Differences in the Plant Senescence Reflectance Index (PSRI) among the trees sampled during behavioral observations. Fig B. Pearson's correlation coefficients between estimates of the tree Decay Index Ratio (DIR). Table C. Summary of foraging routes followed by Magellanic woodpeckers recorded within forest patches. Fig C. Distribution of tree selection probability (W) for the trees used and not used by Magellanic woodpeckers. Fig D. Distribution of residence times (min) for the trees used by Magellanic woodpeckers. Fig E. Distribution of travel distances (m) between trees used by Magellanic woodpeckers. (DOCX) [file pone.0159096.s001.docx]

| **Table A.** Main behaviors elicited by Magellanic woodpeckers according to Short (1970) and classified into two main behavioral states (see main text). Prevalence refers to the percentage of focal observations (number of trees) in which woodpeckers were observed displaying a particular behavior. | | | |
| --- | --- | --- | --- |
| State | Behaviour | Prevalence (%) | Description |
| Non-foraging | Vocalization | 5.4 | Calls and songs |
|  | Drumming | 2.9 | Double tap, used as a signal function. Functionally equivalent to "Drumming" in other woodpecker species. |
|  | Grooming | 4.0 | Performing grooming activities and sometimes stretching. |
|  | Flying | 19.1 | Flights from a section to another within the same tree. |
|  | Observing / Resting | 8.3 | Observing to contiguous trees and/or sections of the same tree. / Inactivity of the individual. Usually, it includes stretching wings while resting. |
| Foraging | Walking | 81.6 | Walks on the ground while inspecting fallen branches or logs. |
|  | Short probing | 76.2 | Briefly probing while moving through a tree. |
|  | Probing / pecking | 51.0 | Characterized by barely audible scraping noises and/or pecking. |
|  | Tapping | 0.4 | Loud repetitive taps made from hard blows with the bill. |
|  | Eating / caching | 8.3 | Successful feeding on wood-boring larvae in a live or dead section of a tree, after performing probing or repetitive taps. |

| **Table B.** Coefficients, standard errors (SE) and p-values from an ordinal regression model evaluating the contribution of the Plant Senescence Reflectance Index (*PSRI*, see text) to the observed decay state of the trees used by Magellanic woodpeckers. Coefficients associated to the overall effect of *PSRI* and threshold parameters for pairwise comparisons between levels of the tree decay state are shown (see Fig. 1S) | | | | |
| --- | --- | --- | --- | --- |
| Effect | Estimate | SE | Z | p |
| Level 1 vs. Level 2 | -8.57 | 1.03 | -8.29 | 0.0000 |
| Level 2 vs. Level 3 | -6.89 | 0.93 | -7.43 | 0.0000 |
| Level 3 vs. Level 4 | -4.93 | 0.80 | -6.19 | 0.0000 |
| Level 4 vs. Level 5 | -2.96 | 0.75 | -3.95 | 0.0001 |
| Overall PRSI effect | 4.10 | 0.55 | 7.52 | 0.0000 |

**Fig A.** Differences in the Plant Senescence Reflectance Index (PSRI) among the trees sampled during behavioral observations and that were classified according into five levels of decay state, following categories provided by Vergara and Schlatter (2004).


**Fig** **B.** On the left: Plot of Pearson's correlation coefficients (r) for estimates of the tree Decay Index Ratio (*DIR*). As explained in the main text, *DIR* were estimated by using the plant senescence reflectance index (PSRI) measured at the tree level. Thus, *DIR* correspond to the ratio between the *DI* value of each tree and the mean *DIR* estimated on the tree that were available over the last four foraging steps (i.e., lags= 0, 1,...,4) as well as along the foraging route (see the main text). On the right: the same correlation matrix plot, but using residuals of *DIR* obtained after regressing the *DIR* for the foraging route on *DIR* at each period lag. Note that *r* values are close to zero when residuals are used whereas for untransformed DIR values, r values are ≥ 0.67.

| **Table C.** Summary of foraging routes followed by Magellanic woodpeckers recorded within forest patches. For each foraging route, it is included the individual woodpecker, the number of foraging trees used by woodpeckers, the mean number of foraging trees available around the foraging trees (located within the 90% isopleth for the circular distribution generated using a BBMM; see text) and summed over the complete route. | | | | |
| --- | --- | --- | --- | --- |
| Route | Individual | Foraging trees (n) | Total available trees | Available trees per step |
| 1 | W1 | 10 | 133 | 13.3 |
| 2 | W1 | 8 | 104 | 13.0 |
| 3 | W2 | 6 | 64 | 10.7 |
| 4 | W2 | 9 | 113 | 12.6 |
| 5 | W3 | 7 | 88 | 12.6 |
| 6 | W3 | 9 | 90 | 10.0 |
| 7 | W3 | 7 | 71 | 10.1 |
| 8 | W4 | 21 | 279 | 13.3 |
| 9 | W4 | 10 | 91 | 9.1 |
| 10 | W4 | 14 | 187 | 13.4 |
| 11 | W5 | 19 | 263 | 13.8 |
| 12 | W5 | 33 | 301 | 9.1 |
| 13 | W5 | 22 | 267 | 12.1 |
| 14 | W6 | 7 | 64 | 9.1 |
| 15 | W7 | 16 | 213 | 13.3 |
| 16 | W7 | 10 | 122 | 12.2 |
| 17 | W7 | 9 | 82 | 9.1 |
| 18 | W7 | 13 | 114 | 8.8 |
| 19 | W8 | 10 | 98 | 9.8 |
| 20 | W7 | 11 | 164 | 14.9 |
| 21 | W8 | 6 | 59 | 9.8 |
| 22 | W8 | 7 | 69 | 9.9 |
| 23 | W9 | 17 | 227 | 13.4 |
| 24 | W9 | 13 | 121 | 9.3 |
| 25 | W9 | 9 | 92 | 10.2 |
| 26 | W10 | 6 | 86 | 14.3 |
| 27 | W10 | 8 | 111 | 13.9 |
| 28 | W11 | 7 | 70 | 10.0 |
| 29 | W11 | 10 | 129 | 12.9 |
| 30 | W11 | 8 | 69 | 8.6 |
| 31 | W11 | 12 | 118 | 9.8 |
| 32 | W11 | 10 | 88 | 8.8 |
| 33 | W12 | 9 | 83 | 9.2 |
| 34 | W13 | 8 | 79 | 9.9 |
| 35 | W13 | 10 | 121 | 12.1 |
| 36 | W13 | 10 | 97 | 9.7 |
| 37 | W14 | 6 | 49 | 8.2 |
| 38 | W14 | 6 | 82 | 13.7 |
| 39 | W14 | 8 | 56 | 7.0 |

**Fig C.** Distribution of Bayesian estimates of the tree selection probability (*W*) for the trees used and not used by Magellanic woodpeckers.

**Fig D.** Distribution of residence times (min) for the trees used by Magellanic woodpeckers.

**Fig E.** Distribution of travel distances (m) between trees that were consecutively used by Magellanic woodpeckers.
